# ﻿*Gymnemaphuquocense* (Apocynaceae, Asclepiadoideae), a new species from Vietnam

**DOI:** 10.3897/phytokeys.251.136457

**Published:** 2025-01-27

**Authors:** Thu Ha Bui, Ngoc Han Le, The Bach Tran

**Affiliations:** 1 Faculty of Biology, Hanoi National University of Education, 136, Xuan Thuy, Cau Giay, Ha Noi, Vietnam Hanoi National University of Education Ha Noi Vietnam; 2 Department of Botany, Institute of Ecology and Biological Resources, Vietnam Academy of Science and Technology, 18 Hoang Quoc Viet, Cau Giay, Ha Noi, Vietnam Institute of Ecology and Biological Resources, Vietnam Academy of Science and Technology Ha Noi Vietnam; 3 Graduate University of Science and Technology, Vietnam Academy of Science and Technology, 18 Hoang Quoc Viet, Cau Giay, Ha Noi, Vietnam Graduate University of Science and Technology Ha Noi Vietnam

**Keywords:** Asclepiadoideae, *Gymnema*, Phu Quoc, Vietnam

## Abstract

A new species of *Gymnema* from Vietnam – *G.phuquocense* – is described, illustrated, and compared with the similar *G.yunnanense*. *Gymnemaphuquocense* differs from *G.yunnanense* by the length of peduncle (3–4 mm vs. 10–13 mm), hairs on corolla lobe margin (absent vs. present), shape of seed (broadly ovate vs. ovate-oblong), shape of scale with 2 prominent longitudinal ridges on corolla tube (lanceolate vs. linear), and length of seeds (9–10 mm vs. 13–15 mm). A diagnostic key of the *Gymnema* species in Vietnam is also provided.

## ﻿Introduction

*Gymnema* R.Br. was established in 1810. The genus comprises approximately 52 species, mainly distributed in tropical or subtropical Asia, S Africa, and Oceania (Li et al. 1995; POWO 2024). *Gymnema* belongs to Apocynaceae-Asclepiadoideae, and is part of the tribe Marsdenieae (Endress and Bruyns 2000). Since more and more species of Marsdenieae became molecularly analysed, the size of the genus *Gymnema* has increased, as e.g. many species of the genus *Marsdenia* (Forster 2021) and *Jasminanthes* (Liede-Schumann et al. 2022) were transferred to *Gymnema*. The genus generally consists of lianas, flowers with the corolla tube with 5 scales (5 prominent longitudinal ridges representing corolline corona formings acc. Liede and Kunze 1993), pollinia erect; fruits with a single follicle developing bearing numerous seeds (Li et al. 1995; Tran 2017). In Vietnam, 7 species of *Gymnema* have been recorded so far (Costantin 1912; Pham 1993; Li et al. 1995; Tran 2005; Tran 2017; POWO 2024).

In March 2022, the authors saw a *Gymnema* species in An Thoi commune, Phu Quoc district, Kien Giang province in Vietnam. After a literature review as well as comprehensive morphological character analysis, we finally confirmed that the species is new to science and thus describe and illustrate it here.

## ﻿Materials and methods

The morphology of the new species was observed on both living plants at the field and herbarium specimens. Branches, leaves, flowers and fruits (lf., fl., fr.) of type materials are stored at the Institute of Ecology and Biological Resources (HN) and the Institute of Tropical Biology (VNM) (acronyms follow Thiers 2024). The conservation status of the new species was assessed according to the guidelines of the International Union for Conservation of Nature (IUCN 2019). Other specimens of *Gymnema* species are studied at HN, VNM and NIMM herbaria that preserved many specimens of species distributed in Vietnam (acronyms follow Thiers 2024).

## ﻿Taxonomy

### ﻿ Gymnema phuquocense

Taxon classificationPlantaeGentianalesApocynaceae

T.B.Tran & T.H.Bui sp. nov.

7E469253-D42C-5B7B-AE17-4BCDA0ED3212

urn:lsid:ipni.org:names:77355778-1

Figs 1 , 2 , 3

#### Type.

Vietnam. • Kien Giang province: Phu Quoc district, An Thoi commune, 28 March 2022, 10°3'32.926"N, 103°59'52.867"E (lf., fl., fr.), *Tran The Bach et al. Bach 28032022-1* (holotype: HN80492!; isotypes: HN80493!, HN80494!, HN80495!, HN80496!, VNM00071049!).

#### Diagnosis.

*G.phuquocense* differs from *G.yunnanense* by the length of peduncle (3–4 mm vs. 5–6 mm), hairs on corolla lobe margins (absent vs. present), shape of seed (broadly ovate vs. ovate-oblong), and shape of corolline corona scales with 2 prominent longitudinal ridges on corolla tube (lanceolate vs. linear).

**Figure 1.**
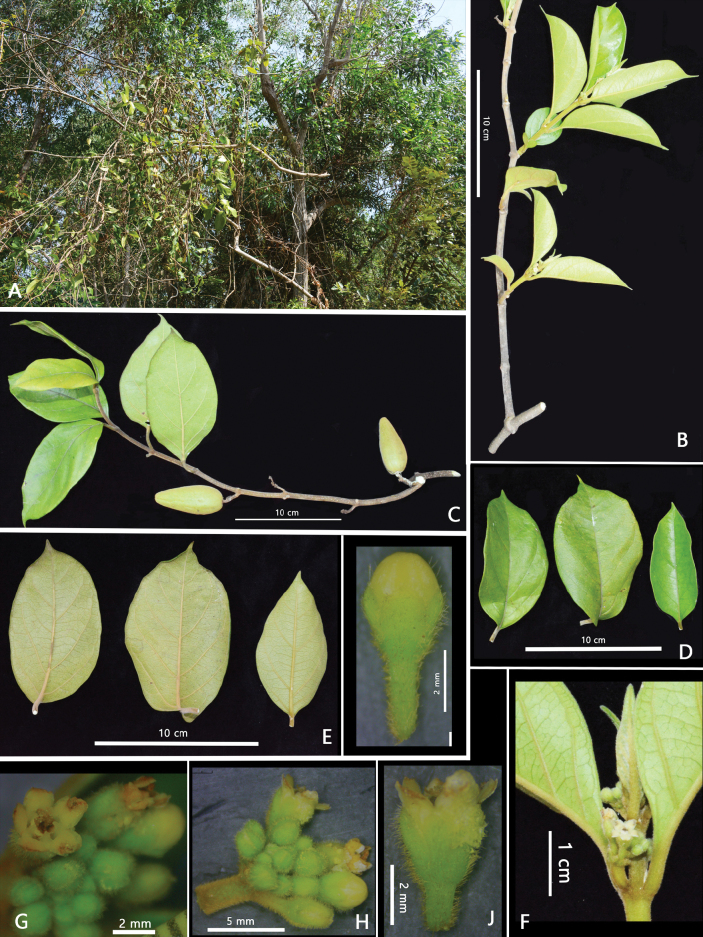
*Gymnemaphuquocense* T.B.Tran & T.H.Bui **A** habitat **B** flowering branch **C** fruiting branch **D** leaf, adaxial surface **E** leaf, abaxial surface **F, G, H** inflorescence **I** flower bud **J** flower (Photos by A.T.Vu, T.B.Tran).

#### Description.

***Lianas***, 10 m or more. Stem pubescent with white trichomes; internodes 2.5–7.5 cm long. ***Leaves*** opposite; petiole 0.5–1.0 cm long, 2.8–3.5 mm diam., pubescent; blade elliptic, 8.8–10.3 cm long, 4.3–6.5 cm wide; adaxial surface glabrous except pubescent nerves, abaxial surface pubescent; base rounded to acuminate; apex acuminate; lateral veins in 4 pairs, retinerved. ***Inflorescences*** extra-axillary, 9–10 mm wide, umbelliform, 16–20-flowered; peduncle 3–4 mm long, pubescent. ***Flowers*** with buds spherical-elliptic, 2–2.5 mm diam., 3.2–4 mm in diam. when anthetic; pedicel with erect hairs, 1.5 mm long, 0.7–1 mm in diam. ***Sepals*** broadly ovate, 1.7–1.8 mm long, ca. 1.4 mm wide; glabrous inside, outside hirsute, margin ciliate, apex rounded, with triangular colleters at the base, 0.34 mm long, 0.23 mm wide;. ***Corolla*** campanulate, inside glabrous, outside with sparse hairs; corolla lobes triangular, ca.1.2 mm long, ca.1.3 mm wide, yellowish; corolla tube with 5 prominent longitudinal scales (=corolline corona) in the sinuses of corolla lobes; scales lanceolate with one line of brownish hairs along each side, apex triangular, white, glabrous, .4–1.6 mm long, 0.6–0.7 mm wide, below part of scale rectangular, ca. 1 × 0.5 mm,. ***Pollinia*** erect, rectangular-elliptic, 0.31 mm long, 0.13 mm wide; corpusculum rectangle-shaped, 0.16–0.17 mm long, 0.06–0.07 mm wide; caudicles 0.02 mm long, 0.05 mm wide. ***Style head*** hemispherical, 0.8–0.9 mm high, 1.2–1.3 mm wide. ***Ovary*** bi-carpellate. ***Follicle* 1**, developed from only one of the two carpels, lanceolate, 5–6 cm long, 2.2–2.5 cm wide; outside pubescent; pericarp 0.9–1.1 mm thick; mature fruits yellow. ***Seeds*** numerous, ca. 60 per follicle; seeds flattened, broadly ovate, 9–10 mm long, 5–6 mm wide, comma 25–30 mm long, white.

**Figure 2.**
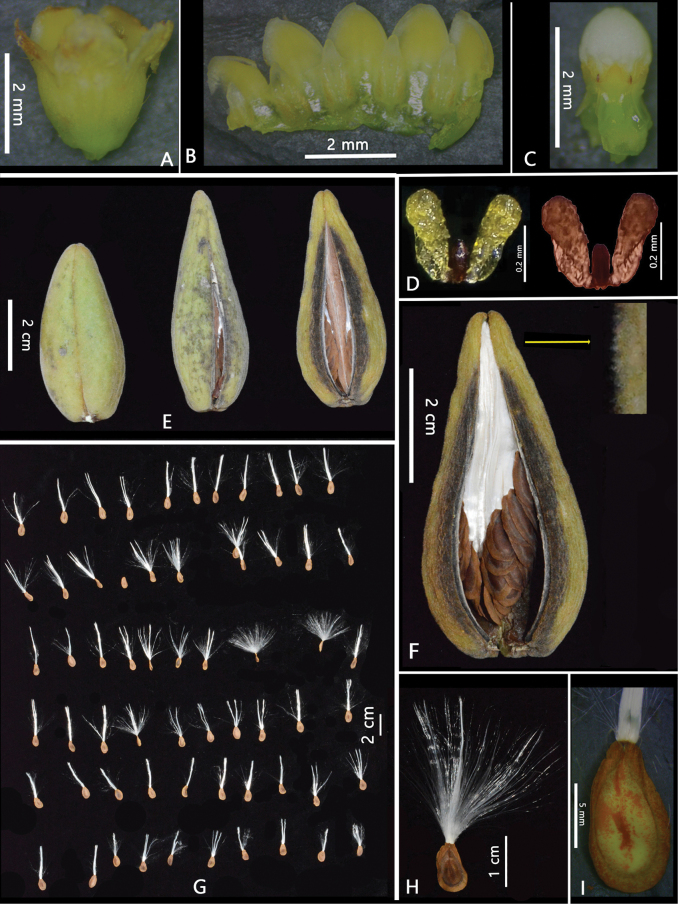
*Gymnemaphuquocense* T.B.Tran & T.H.Bui **A, B** corolla **C** gynostegium **D** pollinarium **E, F** fruit **G, H, I** seeds (Photos by A.T.Vu, T.B.Tran).

#### Etymology.

The specific epithet refers to the type locality, Phu Quoc district in Vietnam.

#### Distribution and ecology.

*Gymnemaphuquocense* was found growing in bright places at the secondary forest of Phu Quoc district, in association with *Acaciaauriculiformis* A.Cunn. ex Benth., *Chromolaenaodorata* (L.) R.M.King & H.Rob., *Micromelumminutum* (G.Forst.) Wight & Arn., *Mimosapudica* L., *Parinariannamensis* Hance, *Psychotriaasiatica* L., *Sidarhombifolia* L., *Stachytarphetajamaicensis* (L.) Vahl, *Streptocaulonjuventas* (Lour.) Merr.,and *Tetracerascandens* (L.) Merr.. Flowering and fruiting were observed in March-April.

**Figure 3.**
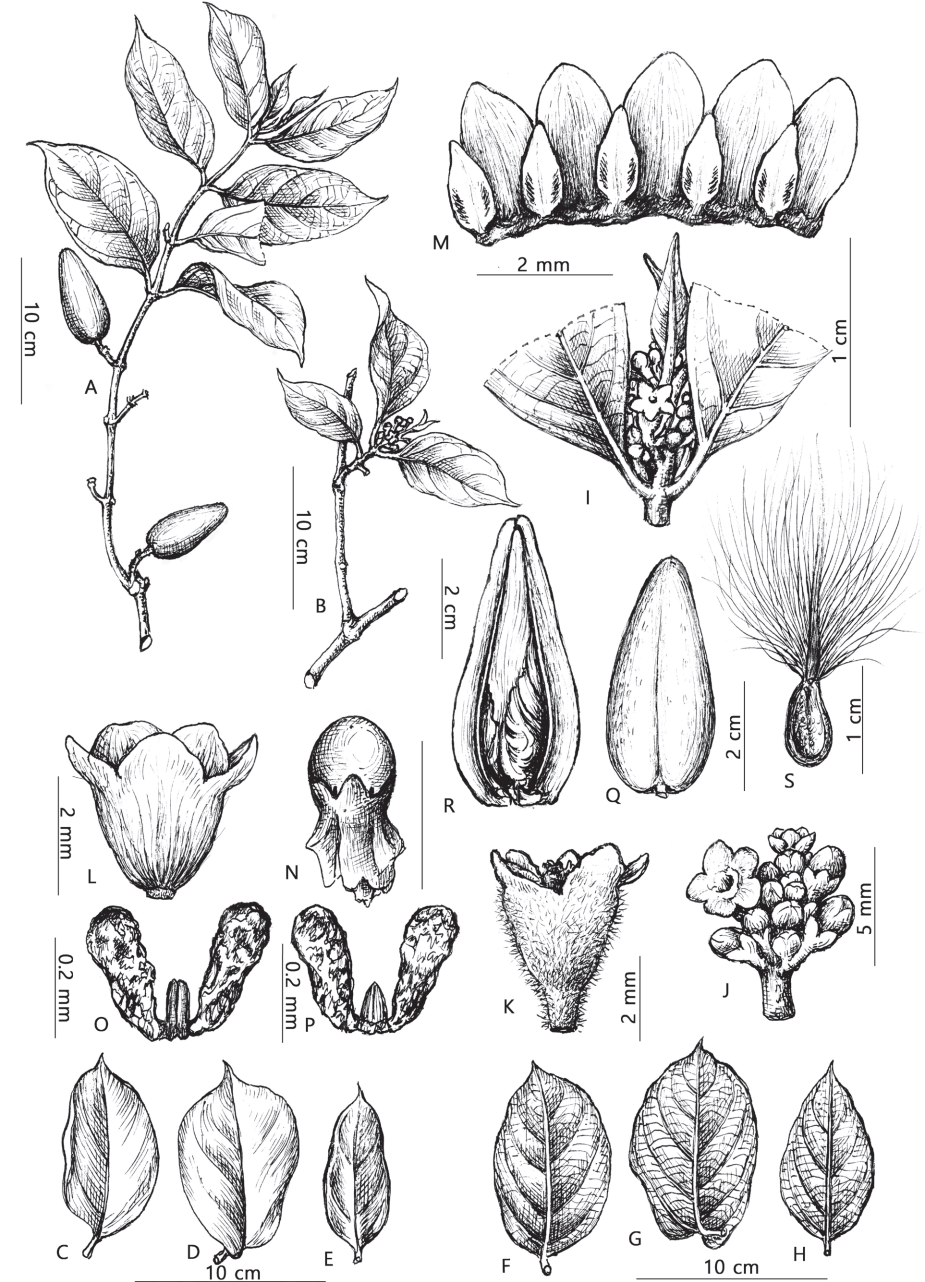
*Gymnemaphuquocense* T.B.Tran & T.H.Bui **A** fruiting branch **B** flowering branch **C, D, E** leaf, adaxial surface **F, G, H** leaf, abaxial surface **I, J** inflorescence **K** calyx **L** corolla **M** 5 scales on corolla **N** gynostegium **O, P** pollinarium **Q, R** fruit **S** seed (Drawn by Le Kim Chi).

#### Conservation status.

Known from only one locality; the preliminary conservation status of *Gymnemaphuquocense* is Data Deficient (DD; IUCN 2019). More fieldwork still need to be done in order to get a better understanding of the full natural distribution of this taxon.

#### Discussion.

The differences between *Gymnemaphuquocense* and *G.yunnanense* are clear (Table 1). *G.yunnanense* is widely distributed on the mainland from SE. Bangladesh to China (S. Yunnan, SW. Guangxi) and Vietnam (Kon Tum province, Kien Giang province: Ha Tien) (Tran 2017, POWO 2024) while *G.phuquocense* is only found on Phu Quoc island (Kien Giang province). However, *Gymnema* species, especially of the *G.sylvestre* group to which the new species belong, are difficult to distinguish. The *G.sylvestre* group includes *G.muelleri* Benth. (Northern Territory, Queensland), *G.tricholepis* Schltr. (New Caledonia, New Guinea, Queensland), *G.stramineum* (P.I.Forst.) P.I.Forst. (Queensland), *G.sylvestre* (Retz.) R.Br. ex Sm. (Tropical & Subtropical Old World), *G.pachyglossum* Schltr. (Philippines), *G.maingayi* Hook.f. (Malaya, Myanmar, Thailand), *G.latifolium* Wall. ex Wight (India to S. China and and Indo-China) and *G.longipedicellatum* (P.I.Forst.) P.I.Forst. (Queensland) (Liede-Schumann et al. 2022, POWO 2024). Another species belonging to this group is *G.thorelii* recorded in Laos, *G.thorelii* (Costantin 1912; POWO 2024), also has many similar characteristics. However, *G.phuquocense* differs from *G.thorelii* by the leaf size (8.8–10.3 × 4.3–6.5 vs. 5–6 × 2.8–3.3), leaf surface (pubescent vs. glabrous), flower diameter (3.2–4 mm vs. 2.5 mm) and the indument of corolla lobe margins (without hairs vs. with hairs). *G.phuquocense* differs from other species of *Gymnema* by the following set of characteristics: peduncle 3–4 mm long, corolla tube hairy but corolla lobe margins not cilliate and not purple, corolline corona scales higher than tube, follicles shorter than 4 cm and seeds broadly ovate, 9–10 mm long.

**Table 1.** Morphological differences between *G.phuquocense* and *G.yunnanense*.

Characters	*G.yunnanense*	*G.phuquocense*
Number of lateral veins (pairs)	**5–6**	**4**
Length of peduncle (mm)	**10–13**	**3–4**
Corolla lobes	**Ciliate**	**not ciliate**
Shape of corolline corona	**Linear**	**Lanceolate**
Shape of seed	**ovate-oblong**	**broadly ovate**
Length of seed (mm)	**13–15**	**9–10**

##### ﻿Key to the species of *Gymnema* in Vietnam

**Table:** 

1	Corolla (excluding “corolline corona”) glabrous	**2**
–	Corolla (excluding “corolline corona”) hairy	**3**
2	Seeds 12.5–14 × 8–9 mm	***G.acuminatum***
–	Seeds ca. 8 × 4 mm	***G.sylvestre***
3	Corolla margins purple; fruit 4–6 cm wide	***G.griffithii***
–	Corolla margins white or yellow; fruit less than 4 cm wide	**4**
4	Corolline corona scales shorter than tube	**5**
–	Corolline corona scales longer than tube	**7**
5	Corolla lobes densely pubescent inside	***G.latifolium***
–	Corolla lobes glabrous inside	**6**
6	Petiole 2–6 cm long; seeds ca. 1.5 × 1 cm, comma ca. 4 cm long	***G.inodorum***
–	Petiole 0.5–1 cm long; seeds ca.1.4 × 0.6 cm, comma ca. 3 cm long	***G.foetidum***
7	Peduncle 10–13 mm long; corolla lobes cilliate; seeds ovate-oblong, 13–15 mm long	***G.yunnanense***
–	Peduncle 3–4 mm long; corolla lobes not cilliate; seeds broadly ovate, 9–10 mm long	***G.phuquocense***

##### ﻿Additional specimens examined

*G.acuminatum*: Vietnam. Dong Nai: Trang Bom, 28 May 1919, Poilane 40886 (VNM); *G.foetidum*: Vietnam, Dak Lak: Krong Pac, 1 June 1979, N. T. Ban, Ban 365 (HN); *G.griffithii*: Vietnam. Dak Lak: Ea H’leo, Ea Sol, 8 August 2011, Tran The Bach, VK 4676 (HN); *G.inodorum*: Vietnam. Hoa Binh, Do Dang Ly 584A, 584B, 584BC, 584D (NIMM); *G.latifolium*: Vietnam. Ninh Binh: Cuc Phuong, NMC 1194, MVX 151, NMC 1321, MVX 658 (HN) – Quang Tri: Huong Hoa, 16 March 2014, Tran The Bach, VK 5880 (HN) – Thanh Hoa, 15 October 1997, VN 305 (HN); *G.sylvestre*: Vietnam. Bac Giang, 6 November 1940, Petelot 2435 (VNM) – Ninh Thuan: Phan Rang, 2 March 1924, Poilane 9856 (VNM), 4 March 1923, Poilane 5563 (VNM)– Quang Ninh, 22 August 2002, V. X. Phuong 5406 (HN); 22 August 2002, V.X. Phuong 5479 (HN); 22 August 2002, V.X. Phuong 5482 (HN); 22 August 2002, V.X. Phuong 5510 (HN); Tien 213 (HN) – Quang Tri: Gio Linh, 13 May 2007, Tran The Bach, VK 1060 (HN) – Vinh Phuc, V. X. Phuong 1061 (HN), T. D. Ly 217 (HN) – Thua Thien Hue: Lang Co, 199 (VNM); *G.yunnanense*: Vietnam. Kien Giang, V. X. Phuong 10124 (HN) – Kon Tum: Dak Glei, VH 1418, (HN), 2 April 2009, Tran The Bach, VK 2593 (HN).

## Supplementary Material

XML Treatment for Gymnema phuquocense

## ﻿Additional information

### Author ORCIDs

Thu Ha Bui https://orcid.org/0000-0001-6971-0105

Ngoc Han Le https://orcid.org/0000-0003-2657-5091

The Bach Tran https://orcid.org/0000-0003-3798-5969

### Data availability

All of the data that support the findings of this study are available in the main text.
